# DeepKinome: quantitative prediction of kinase binding affinity by a compound using deep learning based regression model

**DOI:** 10.3389/fmolb.2025.1698891

**Published:** 2025-12-17

**Authors:** Yeeun Lee, Jisu Eun, Jinhyuk Lee, Seungyoon Nam

**Affiliations:** 1 Department of Genome Medicine and Science, Gachon Institute of Genome Medicine and Science, Gachon University Gil Medical Center, Gachon University College of Medicine, Incheon, Republic of Korea; 2 Bio-design Editing Research Center, Korea Research Institute of Bioscience and Biotechnology (KRIBB), Daejeon, Republic of Korea; 3 Department of Bioinformatics, KRIBB School of Bioscience, University of Science and Technology (UST), Daejeon, Republic of Korea; 4 Department of Health Sciences and Technology, Department of Translational-Clinical Medicine, Gachon Advanced Institute for Health Sciences and Technology (GAIHST), Gachon University, Incheon, Republic of Korea

**Keywords:** kinase activity, kinase inhibition prediction, small molecules, deeplearning, explainable artificial intelligence

## Abstract

**Introduction:**

Kinases are essential for cellular regulation and drug development. Predicting the quantitative binding affinity between small-molecule compounds and kinases remains a challenge because of data complexity.

**Method:**

We developed DeepKinome, a 20-layer convolutional neural network-based deep learning (DL) regression model, to predict quantitative binding affinity. Given the continuous nature of binding affinity, the root mean square error (RMSE), the coefficient of determination (R^2^), the Pearson’s correlation coefficient (PCC) between actual and predicted values, and the acceptance interval ratio (AIR) were evaluated. Trained using data from 234 kinases and 163 compounds from the L1000 database.

**Results:**

DeepKinome outperformed five DL and four machine learning models, achieving an RMSE of 1.157, an R^2^ of 0.535, a PCC of 0.743, and an AIR of 0.570. Explainable artificial intelligence analysis revealed key amino acid sequences that influenced the predictions aligned with known kinase phosphorylation sites.

**Conclusion:**

DeepKinome offers a promising approach for understanding kinase inhibition and compound binding.

## Introduction

Kinases, which comprise the largest enzyme family in the human genome, play a pivotal role in regulating cellular functions through phosphorylation, a reaction that is deeply intertwined with human metabolism (Abdelbaky et al., 2021). Recent advances in our understanding of cell signaling mechanisms have revealed the critical involvement of kinases in oncogenic and metastatic processes (Bhullar et al., 2018). Kinase dysfunctions have been linked to various malignancies driven by genetic mutations and chromosomal reshuffling (Futreal et al., 2004). This has led to an upsurge in kinase-targeted drug research, with over 70 new kinase drugs approved by 2021 (Cohen et al., 2021).

Traditional enzymatic assays measure the enzymatic activity of (Goddard and Reymond, 2004) by incubating a purified active kinase with a substrate and adenosine triphosphate (ATP), followed by detection of phosphorylation (Goddard and Reymond, 2004). The primary focus has been on the functional output (phosphorylation) of kinases (Trenker and Jura, 2020). Functional kinase assays are preferred for screening kinase inhibitors (Goddard and Reymond, 2004). However, these assays are expensive, time-consuming, and technically demanding when scaled to high-throughput formats (Goldstein et al., 2008).

The quick high-throughput screening (HTS) platforms, which measure inhibitor binding independent of enzyme activity, have gained popularity owing to their economic feasibility and technical convenience (Goldstein et al., 2008). Inhibitor-binding-based assays have emerged as valuable alternatives to traditional enzymatic assays (Grant, 2009; Ma et al., 2008). Both enzymatic assays and HTS provide valuable insights into enzymatic activity and inhibitor interactions, respectively; however, they do so in complementary ways. In the early stages of drug discovery, broad-target profiling is often conducted using competition-based HTS (Brooks et al., 2004). Subsequently, enzymatic assays are used to evaluate the functional effects more precisely.

Competition-based HTS is a popular HTS platform that measures the quantitative binding affinity of small molecules to a large panel of kinases (often hundreds of different kinases) (Ma et al., 2008; Blay et al., 2020). It does not measure enzymatic activity directly but instead assesses how well a molecule can bind to different kinases, typically using a competitive binding assay with immobilized kinases and known ligands (Blay et al., 2020). The HTS provides a “binding profile” for a small molecule across a broad spectrum of kinases. It tells you which kinases a molecule can bind to, to some extent, and is useful for understanding the selectivity of kinase inhibitors.

Although the binding affinity from competition-based HTS is a continuous value, no deep learning (DL) studies have directly predicted these continuous values using regression models. Existing artificial intelligence (AI) methods have focused on predicting binary outcomes, such as binding or non-binding, often derived from protein chemical X-ray crystallography structures deposited in the Protein Data Bank (PDB) (Berman et al., 2000). These binary classification models fail to provide information about the strength of the binding affinity (Abdelbaky et al., 2021; Li and Lai, 2007). However, in the era of large-scale HTS data, where binding affinity is provided as a continuous value, there is a pressing need to develop DL models capable of predicting kinase-inhibitor binding affinity. Developing such models to predict the quantitative interactions of small-molecule compounds with kinases is challenging and remains an area of ongoing research because of the nonlinearity of the continuous values of kinase activity inhibition and the high-dimensional nature of the data. Several DL–based approaches have been proposed for quantitative prediction of kinase or drug–target affinity, including DeepDTA (Ozturk et al., 2018), GraphDTA (Nguyen et al., 2021), and DeepIC50 (Joo et al., 2019). DeepDTA employed a 1D convolutional neural network (CNN) to process raw amino-acid and SMILES sequences, achieving reasonable prediction accuracy for dissociation constant (Kd) values but lacking structural interpretability (Ozturk et al., 2018). GraphDTA introduced graph neural networks (GNNs) to encode molecular topology, improving compound representation; however, it still treated protein sequences as unstructured text and did not account for kinase-pocket specificity (Nguyen et al., 2021). DeepIC50 used mutation information and PaDEL 2D molecular descriptors in a CNN regression framework but provided limited biological interpretability (Joo et al., 2019).

In response to this need for quantitative measures of kinase-inhibitor binding affinity, we developed a DL model, DeepKinome, that can accurately and quantitatively predict the binding affinities between kinases and inhibitor compounds. We refer to the percent inhibition (i.e., % inhibition) values obtained from a competition-based HTS (i.e., KINOMEscan) as “binding affinity” for simplicity, although they represent competitive displacement rather than direct binding affinities (i.e., Kd or inhibitory constant (Ki)). This indirect measurement is widely used as a practical surrogate for binding strength in kinase selectivity profiling (Ma et al., 2008; Jacoby et al., 2015; Joisa et al., 2023). By leveraging the growing availability of HTS data, this study provides a novel DL-based approach that moves beyond binary classifications to offer precise quantitative predictions, thereby enhancing the ability to design effective kinase inhibitors.

## Materials and methods

### Data preparation

**FIGURE 1:**
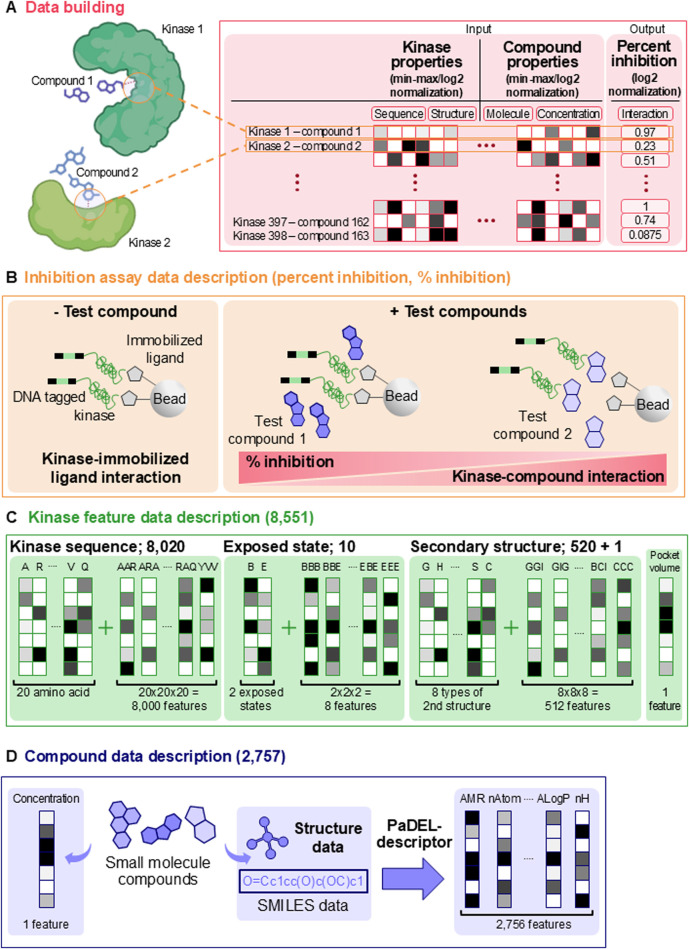
Data description of the study. **(A)** The KINOMEscan high-throughput screening assay that measured the quantitative binding affinity for a kinase-compound pair is depicted. **(B)** In platforms like KINOMEscan, specific proteins (e.g., kinases) are bound to ligands immobilized on the surface of beads. The platform then evaluates whether test compounds bind strongly to the target protein, thereby disrupting the interaction between the protein and the immobilized ligand. In the example illustration, test compound 1 shows a greater percent inhibition measurement (i.e., weaker interaction) compared to test compound 2 because test compound 2 more effectively disrupts the binding between the target protein and the immobilized ligand. **(C)** Description of the kinase features used to train the artificial intelligence models. The 8,551 kinase features are composed of three types of features. **(D)** Description of the compound features for the kinase-compound pairs. 2,757 compound features were calculated using PaDEL software.

DeepKinome directly regresses the binding affinity of a compound-kinase pair on the protein features of the kinase and the chemical descriptors of the compound. The binding affinity was quantified as the percent inhibition of the compound-kinase pair (Figure 1A). Percent inhibition indicates the extent to which a compound binds to kinase active sites, with values ranging from 0% to 100%. A higher percent inhibition represents weaker ligand–kinase displacement (i.e., weaker binding), whereas a lower percent inhibition corresponds to stronger displacement (i.e., stronger binding). This directionality follows the definition used in the KINOMEscan dataset in the “Library of Integrated Network-Based Cellular Signatures” (LINCS) project (Duan et al., 2014). The KINOMEscan assay quantifies ligand–protein competitive displacement, which reflects the ability of a compound to displace a reference ligand from immobilized kinases. Although this approach does not directly measure dissociation constants (e.g., Kd and Ki), the percent inhibition values are widely interpreted as proxies for relative binding affinity across kinase panels (Ma et al., 2008; Jacoby et al., 2015; Joisa et al., 2023).

We obtained 18,771 data points of percent inhibition for kinase-compound pairs involving 234 kinase proteins and 163 kinase inhibitors (small molecule compounds) from the L1000 database (i.e., KINOMEscan high-throughput screening assay data) in the LINCS project (Duan et al., 2014). Reportedly, the percent inhibition data points were obtained by the KINOMEscan® assay (https://www.discoverx.com/home) (Fabian et al., 2005; Duan et al., 2014) (Figure 1B). We also obtained simplified molecular-input line-entry system (SMILES) format data for 163 kinase inhibitors.

The percent inhibition data for the 18,771 kinase-compound pairs were used in a 6:2:2 split for training, validation, and testing (11,253, 3,759, and 3,759 data points, respectively). The data points showed a bimodal distribution skewed towards both extremes (0% and 100%, Supplementary Figure S1). Training the model with an abundance of extreme values (i.e., bimodal distribution) enabled it to adapt excessively to the extreme values (0% and 100%) of the training data. However, the model struggled to adapt effectively to the extreme values in the training data. Therefore, securing the generalization capability of the model to perform well not only on the training data but also on new data is crucial. Excessive optimization of extreme values can lead to a deterioration in the generalization ability of the model. To address these issues during the model training process, we kept the extreme values from the training set unchanged by applying random oversampling (ROS) to the values (range 1%–99%) between the extreme values in the training set. After running the training set using the ROS process, the training set is 18,113. The validation and test sets were not oversampled, leaving them intact.

### Input and output of DeepKinome

The kinase features in the input vector of a kinase-compound pair comprised 8,551 sequence features and molecular properties of the active site of the kinase. The kinase features were divided into four major categories, each representing various protein features calculated for each kinase residue (Figure 1C). The first category was kinase protein sequence features, which included the frequencies of 20 individual amino acids (20 features) and 3-mer amino acid sequences (20 × 20 × 20 features), resulting in 8,020 features (Yang et al., 2018). The sequence of the kinase active site was obtained from the kinase structure in PDB file format by using the Pck software dedicated to the detection and caracterisation of pockets in proteins (Edelsbrunner et al., 1996). The second category includes the secondary structure features of the kinase active site. Each amino acid (i.e., residue) of the kinase active site was assigned to one of the eight secondary structure types (i.e., G, H, I, T, E, B, S, and C) using the “Dictionary of Secondary Structure in Proteins” (DSSP) software (Kabsch and Sander, 1983), and the frequencies of these residues were calculated (eight features). Additionally, in the second category, the frequencies of 3-mer secondary structure sequences were computed (8 × 8 × 8 features), resulting in 520 secondary structure features in the category (Yang et al., 2018). The third category was the kinase protein residue surface exposure state features, which included the frequencies of individual exposed and buried states (two features) as well as the frequencies of 3-mer surface exposure state sequences (2 × 2 × 2 features) (Lee and Richards, 1971). Definition of the exposure states is based on relative water accessible surface area (RSA) values: RSA = (current water accessible surface area)/(ideal water accessible surface area). If the RSA value is ≥ 0.5 the residue is considered exposed, otherwise it is considered buried. The final category is a single feature representing the volume of the compound interaction site (pocket) of the kinase. In summary, the input features for the kinase in a kinase-compound pair amounted to 8,551 features.

The feature data for small-molecule compounds were constructed as input features containing information on 2,756 chemical molecular structures calculated using the PaDEL molecular fingerprint descriptor software (Yap, 2011) from the 2D structure data generated based on the compound’s SMILES format (Figure 1D).

This resulted in 2,756 chemical and molecular structural features of the compounds. Additionally, the concentration information of the compounds used in the experiments was included, resulting in 2,757 elements in the compound molecular property feature data input vector (Figure 1D).

Therefore, the total input vector for the kinase-compound pairs fed into the DL models consisted of 11,308 elements (8,551 kinase features plus 2,757 compound features; Figure 1D).

### DeepKinome model structure

**FIGURE 2:**
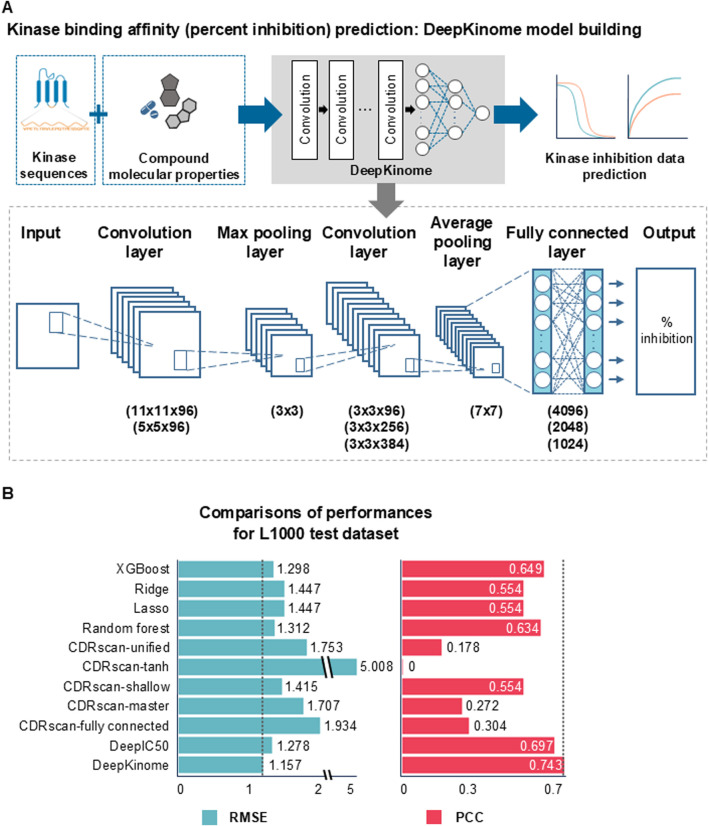
Model overview of DeepKinome and performance comparison. **(A)** Network structure of the DeepKinome model. **(B)** Comparison of the performances of DeepKinome and the other models on the L1000 test dataset. RMSE: root mean square error; AUROC: area under receiver operating characteristic. PCC: Pearson’s correlation coefficients.

DeepKinome is a DL-based regression model that utilizes a convolutional neural network (CNN) to predict compound-induced kinase activity inhibition (i.e., percent inhibition) (Figure 2A). The model architecture consisted of 20 convolutional layers, two pooling layers, and three fully connected layers (Equation 1; Supplementary Table S1). Let the inputs to each convolutional layer, and the output of the layer, the output position index and, the kernel index be represented by *x*, Conv(x)_*ik*_, *i* and *k*, respectively.

$${Conv\left(x\right)}_{ik}=F\left(\sum_{m=0}^{m=M‐1}w_{m}^{k}x_{i+m}+b_{ik}\right)$$ (1)

where *b*_*ik*_ a bias term, w^k^_m_ the *m*th weight in the *k*th kernel tensor, *M* the tensor size, and *F(·)* an activation function (Wang et al., 2021). The activation functions in the model were the Rectified Linear Unit (ReLU) and linear functions. The root mean square propagation optimizer was used for optimization, and the loss function was the root mean squared error (RMSE). The learning rate was set to 1 × 10^−6^, and the model was trained for 150 epochs.

### Performance comparisons with diverse AI models

To validate the performance of AI models, we compared the performance of our proposed method, DeepKinome, with several DL and machine learning (ML) models, including network structures of diverse DL models (CDRScan-fully connected, CDRScan-master, CDRScan-shallow, CDRScan-tanh, CDRScan-unified, and DeepIC50) (Chang et al., 2018; Joo et al., 2019) and ML models (ridge, lasso, XGBoost, and random forest), with CDRScan being a widely used DL network structure for drug screening experimental data prediction. (Chang et al., 2018; Park et al., 2022).

For the DL models, the input layer was modified to match the input data of this study, whereas the network architecture remained the same as that of the original publications (Chang et al., 2018). For DeepIC50 (Joo et al., 2019), the activation function in the last layer was modified from a softmax function to a linear activation function.

The performance evaluation was based on two metrics calculated for the test dataset: Pearson’s correlation coefficient (PCC), which represents the relationship between the actual and predicted values, the coefficient of determination (R^2^), the acceptance interval ratio (AIR), the root mean square error (RMSE), and mean absolute error (MAE). The AIR represents the proportion of predictions falling within a predefined tolerance range (±10%) of the experimental values, providing an intuitive measure of how often the model’s predictions agree closely with real assay data. Higher AIR values indicate a greater proportion of accurate predictions within the predefined tolerance margin (Raicharoen and Lursinsap, 2005).

For the random forest model, we performed grid search optimization to tune the hyperparameters (Ryu et al., 2022). Similarly, the hyperparameters for the lasso, ridge, and XGBoost models were optimized using a grid search (Supplementary Table S2).

We additionally benchmarked DeepKinome using ChemBERTa-based molecular embeddings (Chithrananda et al., 2020) instead of PaDEL 2D descriptors, while keeping the kinase 3-mer features unchanged (henceforth, DeepKinome-ChemBERTa).

### Feature selection

To identify the optimal number of features for the DeepKinome and XGBoost models, we trained the models with varying feature set sizes (2,000, 4,000, 6,000, 8,000, and 10,000) on the training dataset and evaluated their performance on the test dataset using the RMSE. Feature selection using a random forest was performed using the scikit-learn library in Python.

### Explainable artificial intelligence (XAI) for identifying important features in the kinase-compound interaction prediction

In this study, we aimed to understand the features that contribute to the accurate prediction of kinase activity inhibition in kinase-compound pairs. This involves investigating the important features that drive accurate predictions of kinases, for which the DeepKinome method has demonstrated strong performance. To achieve this, we employed an XAI technique, the local interpretable model-agnostic explanation (LIME) method, to assess feature importance.

To identify the features that accurately predicted kinase activity inhibition, we performed the following steps: In the first step, we obtained both the predicted and actual percent inhibition values for all kinase-compound pairs and calculated the PCC between the predicted and actual values. This step assessed how well the predicted percent inhibition values matched the actual observations. In the second step, based on the PCCs, we selected the top five kinases with the highest PCC values. These kinases were considered the most accurate models for predicting the percent inhibitions values of the compounds in our dataset.

## Result

### Overview of study

To predict the quantitative binding affinities between kinases and small-molecule compounds, we developed a DL-based regression model called DeepKinome, which can predict the kinase activity inhibition of kinase-compound interactions by utilizing information on kinase amino acid sequences, binding pockets, and the structural characteristics of small-molecule compounds.

The model uses the molecular and topological information of kinases and small molecule compounds as input, and outputs the predicted percent inhibition of kinase activity upon binding with the compound (Figure 2A). The percent inhibition value represents the degree of binding between the kinase and the compound, with lower values indicating stronger binding and higher values indicating weaker binding.

We also compared DeepKinome with the performances of six other DL models (DeepIC50, CDRscan-master, CDRscan-fully connected, CDRscan-tanh, CDRscan-shallow, and CDRscan-unified) (Chang et al., 2018) and 4 ML models (random forest, lasso, ridge, and XGBoost).

### Inspection on chemical diversity in the dataset

The predictive performance of AI algorithms is intrinsically linked to the diversity of the training data (Ryu et al., 2022). We assessed the diversity of compounds and kinases used to train the models to ensure robust and generalizable predictions. Therefore, we evaluated the chemical diversity of the compounds as an initial step. To assess the diversity of the kinases, we examined the distribution of individual kinases across the overall kinase classification. In addition, t-distributed stochastic neighbour embedding (t-SNE) was used to evaluate compound diversity. These results confirmed that the compounds and kinases used in our study exhibited high chemical diversity and broad distribution across the kinase classification tree. (Supplementary Figure S2A,B).

### Performance evaluation of DeepKinome and other AI models using L1000 test dataset

Consequently, to assess the performance of the 11 regression models for predicting kinase activity inhibition (i.e., percent inhibition), including DeepKinome, the other six DL models (Chang et al., 2018; Joo et al., 2019), and the other 4 ML models after training, we calculated the RMSE and PCC between the observed and predicted percent inhibitions in the test set (Figure 2B). A lower RMSE and higher correlation values indicate better predictive accuracy. The performance of the DeepKinome model on the L1000 test dataset was measured, with an RMSE of 1.157 and a PCC of 0.743.

In this study, ROS was selectively applied to the mid-range region (1%–99%) of the training data to stabilize regression and enhance feature learning in the sparsely represented mid-range region, while keeping the extreme values unchanged. The main purpose of this design is to improve the model’s generalization capability under real-world imbalance conditions by exposing it to a more balanced distribution during training. However, the test set retains the data distribution characteristics as in real-world data (Salmi et al., 2024). For DeepKinome, performance comparison, in the test set, before and after ROS confirmed improved generalization (R^2^ from 0.524 to 0.535; PCC from 0.733 to 0.743), indicating that ROS did not harm learning at the extremes but instead enhanced stability across the mid-range region.

The RMSEs and PCCs of the ML models (ridge, lasso, random forest, and XGBoost) were 1.298–1.447 and 0.554–0.649, respectively. Among the ML models, the random forest and XGBoost models showed superior performance compared with the lasso and ridge models; however, their performance was still less effective than that of DeepKinome. The RMSEs and PCCs of the six DL models were 1.278–5.008 and 0-0.697, respectively. Among the six DL models, DeepIC50 (an RMSE of 1.278 and a PCC of 0.697) showed superior performance compared to the other DL models, but its performance was still lower than that of DeepKinome. DeepKinome also outperformed DeepIC50 in R^2^ and AIR (Supplementary Table S3). Notably, AIRs (0.570 and 0.260 in DeepKinome and DeepIC50, respectively) showed that DeepKinome over DeepIC50 predictions more frequently fall within the acceptable tolerance range.

We additionally benchmarked DeepKinome-ChemBERTa utilizing molecular embedding. The DeepKinome-ChemBERTa model showed an RMSE of 1.322, an R^2^ of 0.450, and a PCC of 0.674. As a result, DeepKinome original architecture (an RMSE of 1.157, an R^2^ of 0.535, and a PCC of 0.743) outperformed DeepKinome-ChemBERTa. We believe this is because biological datasets—unlike image or language data—often lack dense relational structure, limiting the effectiveness of very deep transformer or GNN architectures (Boadu et al., 2023).

Overall, the DeepKinome model demonstrates the best performance among the DL and ML methods. The second-best model was DeepIC50. Also, 3-fold cross validation in the training set showed the significant performance improvement of DeepKinome over DeepIC50 (Supplementary Table S4).

### Performance comparison of DeepKinome and XGBoost according to feature selection

**FIGURE 3:**
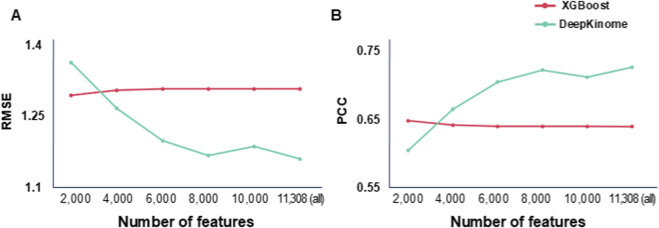
Prediction performance of DeepKinome and XGBoost according to selected features. **(A)** According to selected features, RMSE values of the models in the test set were plotted. Higher RMSE indicates lower prediction accuracy. **(B)** The PCC between the predicted and observed percent inhibition was measured in the test set according to selected features.

We investigated whether feature selection can improve the performance of DeepKinome and XGBoost (the best-performing model among the ML models). To achieve this, we trained the two models on the training set and evaluated their performance on the test set using the RMSE and PCC (Figures 3A,B).

The results showed that DeepKinome achieved the best performance when utilizing all 11,308 features. In contrast, XGBoost exhibited optimal performance when the number of features was 2,000, yielding an RMSE of 1.286 and a PCC of 0.653.

These findings indicate that XGBoost benefits from feature reduction, whereas the DeepKinome model performs the best when leveraging the full feature set. A comparative analysis of the DeepKinome and XGBoost models across different selected features revealed that the DeepKinome model consistently outperformed XGBoost, except 2,000 features level, demonstrating a lower RMSE and higher PCC, indicative of superior predictive performance.

### Feature importance by XAI

**FIGURE 4:**
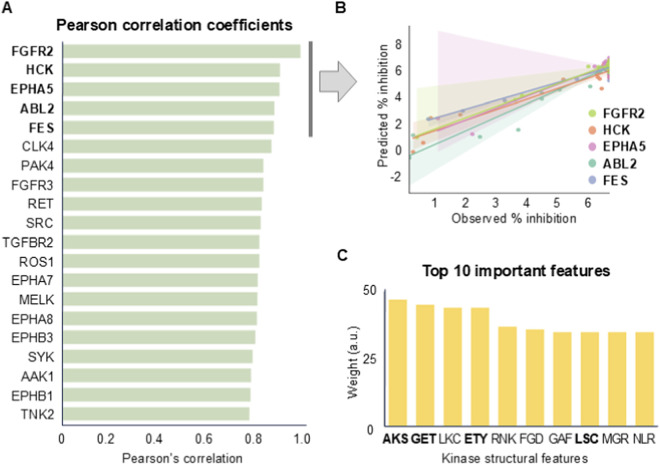
Feature importance analysis by applying explainable artificial intelligence to DeepKinome to identify the features influencing kinase activity inhibition prediction. **(A)** A bar plot illustrating the top 20 kinases with the highest predictive performance achieved by DeepKinome. **(B)** A scatter plot showing the observed and predicted kinase-compound binding affinities for the top 5 kinases (from (A)) with high predictive performance. **(C)** The top 10 features that had the greatest impact on the DeepKinome predictions. The features are 3-mer amino acid sequences.

To identify the features that influenced the DeepKinome model’s prediction of kinase activity inhibition, we ranked the kinases in the test set based on their prediction performance (i.e., PCC) and selected the top five kinases: fibroblast growth factor receptor 2 (FGFR2), hematopoietic cell kinase (HCK), Eph receptor A5 (EPHA5), ABL proto-oncogene 2 (ABL2), and FES proto-oncogene, tyrosine kinase (FES) (indicated by the gray vertical line in Figure 4A). We then applied the XAI technique to the 94 kinase-compound pairs for the top five kinases (Figures 4A,B).

The XAI analysis revealed the top 10 features that contributed the most to the prediction of percent inhibition by the DeepKinome model (Figure 4C). Interestingly, the top 10 features (AKS, GET, LKC, ETY, RNK, FGD, GAF, LSC, MGR, and NLR) were all 3-mers of amino acids, four of which (AKS, GET, ETY, and LSC) contained amino acid residues (S, T, and Y) that are frequently phosphorylated (Miller and Turk, 2016). This suggests that the DeepKinome model places significant importance on phosphorylation-related amino acid sequences when predicting the percent inhibition.

Overall, our XAI-based feature importance analysis indicates that DeepKinome leverages phosphorylation-associated amino acid sequences as key features for the prediction of kinase activity inhibition.

## Discussion

In this study, we introduced DeepKinome, a DL-based regression model designed to predict quantitative kinase activity inhibition by small-molecule compounds. HTS methods have revolutionized the discovery of kinase inhibitors, offering a more scalable and cost-effective alternative to traditional enzymatic assays, which, while reliable, are often resource-intensive and less suitable for large-scale applications (Rudolf et al., 2014). However, most existing computational models in this space are limited to binary predictions, identifying only whether a molecule binds to a kinase without measuring the strength of the binding (Abdelbaky et al., 2021; Li and Lai, 2007). Our study contributes to the accurate prediction of kinase-inhibitor interactions by developing a DL model capable of predicting how strongly a molecule binds to a kinase, rather than just whether it binds.

Kinases play crucial roles in diverse biological pathways and aberrant kinase activity has been associated with numerous diseases (Cohen, 2002). Therefore, designing and improving compounds capable of effectively inhibiting kinases ultimately remains an important endeavor in developing more efficacious therapeutics (Anderson et al., 2023). While current research has primarily focused on the binary classification (i.e., binding and non-binding) of kinase-ligand binding modes using AI approaches (Abdelbaky et al., 2021; Thafar et al., 2019; Miljkovic et al., 2020), studies predicting quantitative kinase activity inhibition are rare (Gagic et al., 2019). Our study demonstrates that DeepKinome outperforms other DL and ML models in terms of lower RMSE and higher PCC. Among the ML models, XGBoost exhibited the best performance, and we investigated whether feature selection could further improve the performance (Figure 3). Although XGBoost showed promising results for this regression problem through feature selection, it did not surpass the performance of the DeepKinome model (Figure 3).

Although DeepKinome achieved near-assay-level accuracy (an RMSE of 1.157 on a 0%–100% scale), we acknowledge that even small percent differences in inhibition can be critical in drug discovery and lead-optimization contexts. This limitation indicates that, while the model performs well for large-scale virtual screening and trend identification, its predictive resolution may not yet be sufficient for precise compound ranking or dose–response optimization. Therefore, the predicted inhibition values should be interpreted with caution in early-stage decision-making, and future improvements will focus on training with larger, higher-resolution datasets to enhance quantitative granularity and predictive precision.

The use of a multi-layer 1D CNN may appear somewhat arbitrary; however, this approach has been widely adopted in kinase- and drug–response prediction studies, including CDRscan (Chang et al., 2018), where CNNs have demonstrated strong performance in learning molecular patterns from tabular or sequence-like features. Considering this established precedent, we maintained the CNN-based architecture over alternative architectures.

It is important to note that ChemBERTa (Chithrananda et al., 2020) learns molecular information from SMILES strings, which are text-like representations of chemical structures. However, the same molecule can often be written in different SMILES sequences, even though its actual 3D structure does not change (David et al., 2020). Because ChemBERTa reads molecules as text rather than as real chemical structures, this can sometimes cause inconsistencies between how a molecule looks and how it is encoded. As a result, models trained only on SMILES tokens may not fully capture the structural features that determine binding affinity, which could partly explain the slightly lower performance of the ChemBERTa benchmark (i.e., DeepKinome-ChemBERTa) compared to the original PaDEL-based DeepKinome.

While external validation using ChEMBL (Mendez et al., 2019) or BindingDB (Liu et al., 2025) was not possible due to the absence of compatible percent inhibition data, internal 3-fold cross-validation in the LINCS/KINOMEscan dataset also yielded better performance of DeepKinome over DeepIC50 across the folds (Supplementary Table S4). This stability supports the robustness of DeepKinome, although future work will focus on testing the model against independent kinase–inhibitor datasets once standardized quantitative binding data become available.

In the present study, we employed DeepKinome to predict the kinase activity and identified the top five kinases (FGFR2, HCK, EPHA5, ABL2, and FES) with the highest prediction accuracy (Figures 4A,B). The feature importance analysis was demonstrated on a subset of five representative kinases (94 compound–kinase pairs) rather than the entire dataset. This subset was intentionally selected to illustrate the interpretability framework using LIME, which provides local instance–level explanations rather than global feature attributions. Because LIME operates on individual prediction instances, it is not feasible to aggregate its weight meaningfully across hundreds of kinases with heterogeneous biochemical contexts. Therefore, our analysis focused on representative kinases (FGFR2, HCK, EPHA5, ABL2, and FES) to capture a wide range of binding mechanisms. The goal was not to generalize feature weights across all kinases but to demonstrate how the model learns biologically relevant 3-mer patterns at the local level.

In addition, based on the literatures, kinases are closely linked to diverse cancer types. FGFR2, a member of the fibroblast growth factor receptor family, plays a crucial role in various biological processes, including cell proliferation, regeneration, and angiogenesis, which are hallmarks of cancer (Turner and Grose, 2010). Mutations in the *FGFR2* often occur in endometrial, non-small cell lung, and gastric cancers (Krook et al., 2021). HCK is a member of the Src family of kinases and is primarily expressed in leukocytes (Luo et al., 2023). HCK is involved in immune and inflammatory responses and has been implicated in various leukocyte-related disorders, particularly chronic myelogenous leukemia (CML) and acute myeloid leukemia (AML) (Lowell, 2011). In an immunotherapy cohort, mutations in *EPHA5* in lung adenocarcinoma resulted in longer progression-free survival than the wild-type (Huang et al., 2021). ABL2 involved in the development of various cancer types by regulating cytoplasmic signaling pathways that influence cell survival, proliferation, and migration (Coluccia et al., 2007). *FES* encodes the tyrosine protein kinase Festival (Fp), a non-receptor tyrosine kinase that is involved in cell proliferation, differentiation, and migration, and is reportedly hyperactivated in cancer (Miyata et al., 2013; Greer, 2002).

The XAI analysis of DeepKinome was performed on the five kinases, and the 10 most important 3-mer amino acid sequences (AKS, GET, LKC, ETY, RNK, FGD, GAF, LSC, MGR, and NLR) were identified. Of the ten sequences, four (AKS, GET, ETY, and LSC) contained amino acid residues (S, T, and Y) that are known to be the preferred phosphorylation sites in kinases (Miller and Turk, 2016), demonstrating that our model accurately reflects the kinase preferences reported in previous studies.

Among the top-ranked sequences, AKS, GET, ETY, and LSC contain amino acids such as serine (S), threonine (T), or tyrosine (Y), which are commonly targeted during phosphorylation. These sequences are therefore likely to represent positions where phosphate groups are directly added (Abdelbaky et al., 2021). LKC, RNK, FGD, GAF, MGR, and NLR do not include these specific residues, but they contain amino acids frequently observed near the active or catalytic sites of kinases (Endicott et al., 2012). For example, positively charged residues such as lysine (K) and arginine (R) can engage in electrostatic interactions with the phosphate groups of ATP, thereby contributing to their stabilization (Endicott et al., 2012). Aspartate (D) may participate in coordinating magnesium ions (Mg^2+^), which are essential for phosphate transfer reactions. Glycine (G) provides backbone flexibility, facilitating structural adjustments around the catalytic pocket. Although these sequences may not directly mark phosphorylation sites, they appear to play roles in shaping the local environment where kinase activity occurs (Endicott et al., 2012; Abdelbaky et al., 2021).

A limitation of our study is that the kinase activity inhibition data exhibited an imbalance with a skewed distribution towards extreme values. To address this limitation in data balance, we implemented a random oversampling approach on the training data to mitigate the data imbalance.

## Conclusion

In this study, we have developed a DL-based regression model, DeepKinome, for predicting the kinase activity inhibition between kinases and small molecule compounds, quantitatively. The model, which used physical and chemical molecular features of kinases and small molecules for prediction, demonstrated better performance over the other DL and ML models.

## Data Availability

Publicly available datasets were analyzed in this study. This data can be found here: https://lincsportal.ccs.miami.edu/dcic-portal/. The source codes are available at GitHub: https://github.com/labnams/DeepKinome.
