# Supplementary material for: DeepKinome: quantitative prediction of kinase binding affinity by a compound using deep learning based regression model
Source: Front Mol Biosci. 2025 Dec 17;12:1698891. doi: 10.3389/fmolb.2025.1698891 (PMC12709132; doi:10.3389/fmolb.2025.1698891)

Supporting Information

DeepKinome: Quantitative Prediction of Kinase Binding Affinity by a Compound Using Deep Learning Based Regression Model

Lee *et al*.

Supplementary Table S1 through S4

Supplementary Figure S1 through S2

Supplementary Table S1. Our implementation of DeepKinome for kinase-compound interaction prediction. The DeepKinome structure uses 20 convolution layers and 3 fully connected layer. The 'module type' column represents that type of specific modules, the 'Param #' column represents number of parameters, respectively.

Layer	Output Shape	Param #
Input Layer	(11308 x 1)	0
Convolution layer 1D_1	(2827 x 96)	1152
Batch normalization layer_1	(2827 x 96)	384
Activation function_1	(2827 x 96)	0
Convolution layer 1D_2	(1414 x 96)	46176
Batch normalization layer_2	(1414 x 96)	384
Activation function_2	(1414 x 96)	0
Max pooling layer	(707 x 96)	0
Convolution layer 1D_3	(707 x 96)	27744
Batch normalization layer_3	(707 x 96)	384
Activation function_3	(707 x 96)	0
Convolution layer 1D_4	(707 x 96)	27744
Batch normalization layer_4	(707 x 96)	384
Activation function_4	(707 x 96)	0
Convolution layer 1D_5	(707 x 256)	73984
Batch normalization layer_5	(707 x 256)	1024
Activation function_5	(707 x 256)	0
Convolution layer 1D_6	(707 x 256)	196864
Batch normalization layer_6	(707 x 256)	1024
Activation function_6	(707 x 256)	0
Convolution layer 1D_7	(707 x 256)	196864
Batch normalization layer_7	(707 x 256)	1024
Activation function_7	(707 x 256)	0
Convolution layer 1D_8	(707 x 256)	196864
Batch normalization layer_8	(707 x 256)	1024
Activation function_8	(707 x 256)	0
Convolution layer 1D_9	(707 x 256)	196864
Batch normalization layer_9	(707 x 256)	1024
Activation function_9	(707 x 256)	0
Convolution layer 1D_10	(707 x 256)	196864
Batch normalization layer_10	(707 x 256)	1024
Activation function_10	(707 x 256)	0

Convolution layer 1D_11	(707 x 384)	295296
Batch normalization layer_11	(707 x 384)	1536
Activation function_11	(707 x 384)	0
Convolution layer 1D_12	(707 x 384)	442752
Batch normalization layer_12	(707 x 384)	1536
Activation function_12	(707 x 384)	0
Convolution layer 1D_13	(707 x 384)	442752
Batch normalization layer_13	(707 x 384)	1536
Activation function_13	(707 x 384)	0
Convolution layer 1D_14	(707 x 384)	442752
Batch normalization layer_14	(707 x 384)	1536
Activation function_14	(707 x 384)	0
Convolution layer 1D_15	(707 x 384)	442752
Batch normalization layer_15	(707 x 384)	1536
Activation function_15	(707 x 384)	0
Convolution layer 1D_16	(707 x 384)	442752
Batch normalization layer_16	(707 x 384)	1536
Activation function_16	(707 x 384)	0
Convolution layer 1D_17	(707 x 256)	295168
Batch normalization layer_17	(707 x 256)	1024
Activation function_17	(707 x 256)	0
Convolution layer 1D_18	(707 x 256)	196864
Batch normalization layer_18	(707 x 256)	1024
Activation function_18	(707 x 256)	0
Convolution layer 1D_19	(707 x 256)	196864
Batch normalization layer_19	(707 x 256)	1024
Activation function_19	(707 x 256)	0
Convolution layer 1D_20	(707 x 256)	196864
Batch normalization layer_20	(707 x 256)	1024
Activation function_20	(707 x 256)	0
Average pooling layer	(354 x 256)	0
Flatten function	(90624)	0
Fully connected layer_1	(4096)	371200000
Activation function	(4096)	0
Fully connected layer_2	(2048)	8390656
Activation function	(2048)	0
Fully connected layer_3	(1024)	2098176
Activation function	(1024)	0
Prediction layer	(1)	1025

Total params: 386,266,785
Trainable params: 386,256,289
None-trainable params: 10,496

Supplementary Table S2. Parameters for machine learning models.

Model	Parameter	Parameter value
random forest	The number of estimators	200
random forest	Max depth	30
random forest	Max feature	sqrt(number of features)
random forest	Min samples leaf	1
random forest	Min samples split	2
ridge	alpha	10
lasso	alpha	0.0004
XGBoost	Max depth	8
XGBoost	Min child weight	0.001
XGBoost	gamma	0.01
XGBoost	learning rate	0.1

Supplementary Table S3. Performance comparisons of DeepKinome with DeepIC50 in the test set.

Model	RMSE	R^2	PCC	AIR	MAE
DeepKinome	1.157	0.535	0.743	0.570	1.488
DeepIC50	1.278	0.432	0.697	0.260	1.312

Supplementary Table S4. Comparative performance of DeepKinome and DeepIC50 models evaluated by 3-fold cross-validation. Summary of mean ± standard error of the mean (SEM) for Pearson correlation coefficient (PCC), coefficient of determination (R^2), and RMSE across the three folds in the training set, with corresponding p-values indicating statistically significant improvement of DeepKinome over DeepIC50. The Welch's t-tests were performed to test significance.

Evaluation metric	DeepKinome	DeepIC50	p-value
PCC	0.730 ± 0.008	0.691 ± 0.001	0.0048
R^2	0.529 ± 0.014	0.459 ± 0.002	0.0044
RMSE	1.183 ± 0.019	1.600 ± 0.016	4.45×10^{-5}

Supplementary Figure S1. Training, validation, and test dataset distribution. Data used to build all models were obtained from LINCS project L1000 database.

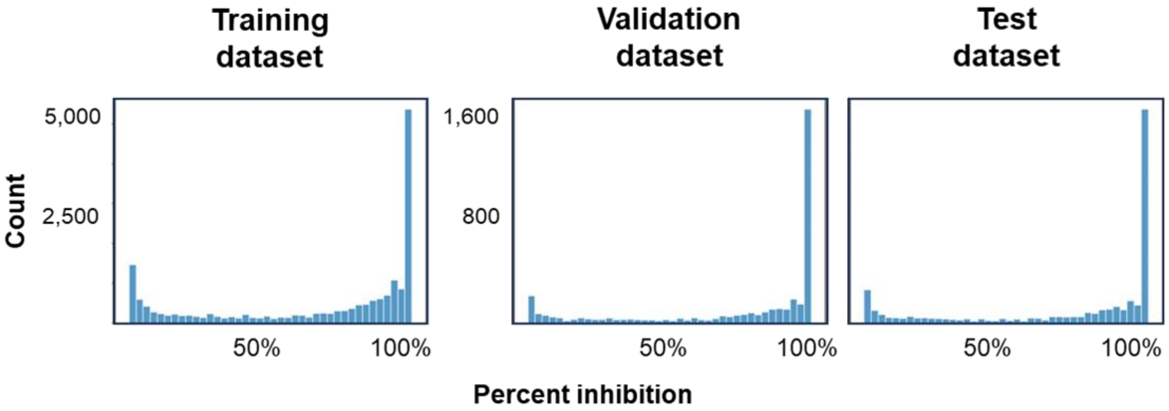

Supplementary Figure S2. Diversity of kinases and compounds. A. The diversity of the kinases. **B**. The diversity of the compounds.

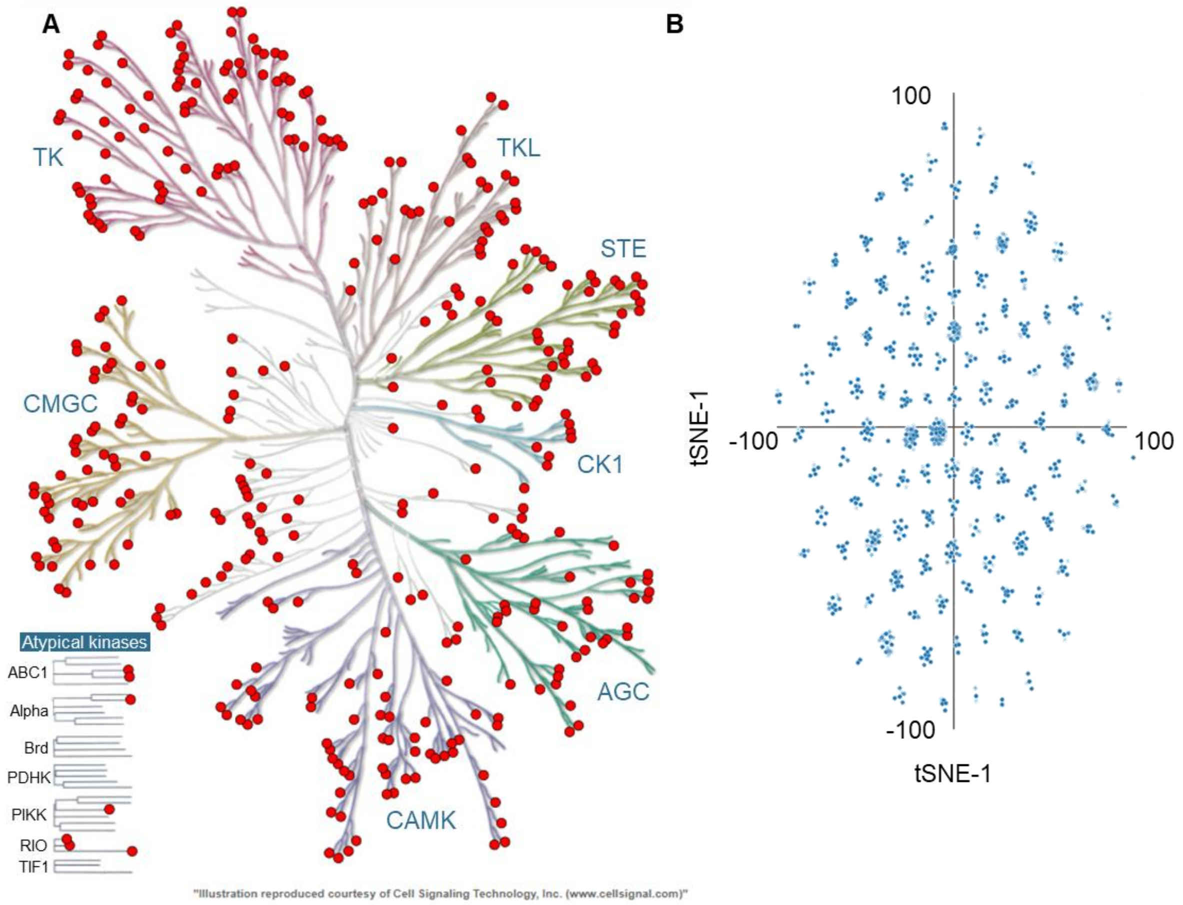

Supplement: Supplementary file 1 [file DataSheet1.pdf]
